# Conditional and Synthetic Type IV Pili-Dependent Motility Phenotypes in *Myxococcus xanthus*

**DOI:** 10.3389/fmicb.2022.879090

**Published:** 2022-05-02

**Authors:** Kalpana Subedi, Daniel Wall

**Affiliations:** ^1^Department of Molecular Biology, University of Wyoming, Laramie, WY, United States; ^2^Department of Chemistry, University of Wyoming, Laramie, WY, United States

**Keywords:** synthetic phenotype, gliding, myxobacteria, exopolysaccharide, type IV pili, *Myxococcus xanthus*, social motility, twitching motility

## Abstract

Myxobacteria exhibit a variety of complex social behaviors that all depend on coordinated movement of cells on solid surfaces. The cooperative nature of cell movements is known as social (S)-motility. This system is powered by cycles of type IV pili (Tfp) extension and retraction. Exopolysaccharide (EPS) also serves as a matrix to hold cells together. Here, we characterized a new S-motility gene in *Myxococcus xanthus*. This mutant is temperature-sensitive (Ts^–^) for S-motility; however, Tfp and EPS are made. A 1 bp deletion was mapped to the MXAN_4099 locus and the gene was named *sglS*. Null mutations in *sglS* exhibit a synthetic enhanced phenotype with a null *sglT* mutation, a previously characterized S-motility gene that exhibits a similar Ts^–^ phenotype. Our results suggest that SglS and SglT contribute toward Tfp function at high temperatures in redundant pathways. However, at low temperatures only one pathway is necessary for wild-type S-motility, while in the double mutant, motility is nearly abolished at low temperatures. Interestingly, the few cells that do move do so with a high reversal frequency. We suggest SglS and SglT play conditional roles facilitating Tfp retraction and hence motility in *M. xanthus*.

## Introduction

Type IV pili (Tfp) play a variety of important roles in the biology of bacteria including motility, adhesion, biofilms, natural competence, and virulence (Wall and Kaiser, 1999; Burrows, 2012; Craig et al., 2019). Over the past five decades, *Myxococcus xanthus* served as a leading model for understanding Tfp function, with research focused on their role in social (S)-motility and multicellular interactions (Kaiser, 1979; Wall and Kaiser, 1999). More recently, cryo-electron tomography studies in *M. xanthus* elegantly revealed the overall architecture of the Tfp machine (Chang et al., 2016; Treuner-Lange et al., 2020). This structure spans the Gram-negative cell envelope and consists of 10 core proteins. Although much is known about Tfp and S-motility, our understanding is incomplete because new genes continue to be discovered. These discoveries are primarily driven by gene candidate approaches. Such strategies recently unraveled the nature of the PilY tip adhesin family and a new biosurfactant involved in S-motility (Islam et al., 2020; Treuner-Lange et al., 2020). In other model systems, fundamental discoveries about Tfp function were also made, where the term twitching motility is typically used in lieu S-motility (Skerker and Berg, 2001; Burrows, 2012; Craig et al., 2019).

S-motility plays central roles in myxobacterial multicellular behaviors that include development, rippling and predation (Mauriello et al., 2010; Muñoz-Dorado et al., 2016). For these reasons, numerous labs study S-motility where genetic analysis showed that Tfp (Wu and Kaiser, 1995; Wall and Kaiser, 1999) and exopolysaccharide (EPS; Li et al., 2003; Lu et al., 2005) are the primary host structures involved. Here, motility is powered by cycles of pili extension and retraction, whereby retraction pulls the cells forward (Skerker and Berg, 2001; Craig et al., 2019). EPS serves as an extracellular matrix that holds cells together and as a cue for pili retraction and cell reversal control (Li et al., 2003; Zhou and Nan, 2017). Additionally, *M. xanthus* contains a second surface motility system, called adventurous (A) motility (Pathak and Wall, 2012), which uses an independent motor to power cell movements (Nan et al., 2011; Sun et al., 2011). A chemosensory-like signal transduction pathway (Frz) and a G-protein polarity switch coordinates the direction of cell movements powered by these two motors (Schumacher and Sogaard-Andersen, 2017).

In contrast to a targeted gene candidate approach, we are interested in an unbiased forward genetic strategy to identify new genes involved in S-motility. In particular, we are interested in a class of mutants generated by chemical mutagenesis whereby cells are defective in S-motility yet retain Tfp and EPS production (Wu et al., 1997). One example of this phenotype are *pilT* mutants, which are defective in motor function for pili retraction (Clausen et al., 2009). Another example are *sglT* (MXAN_3284) mutants, which we recently described (Troselj et al., 2020). These type of mutants offer insights into Tfp function and the control of pili retraction. Consistent with this, a prior study showed that although Δ*pilT* mutants are defective in pili retraction, residual retraction nevertheless still occurs suggesting another protein(s) is involved (Clausen et al., 2009). Additionally, in other Tfp systems involving twitching, there are two ATPase retraction motors, PilT and PilU (Adams et al., 2019; Tala et al., 2019), but in *M. xanthus* only PilT is known. Aside from the retraction motors, the cellular control and external signal(s) governing retraction are not fully understood. Here we describe a new S-motility protein, named SglS, which exhibits similar mutant phenotypes as *pilT* mutants, but are conditional. We additionally show that when a *sglT* null mutation (Troselj et al., 2020), which has similar phenotypes as *sglS* mutants, are combined, the resulting double mutant exhibits an enhanced synthetic S-motility phenotype, suggesting these proteins function in redundant pathways, possibly related to Tfp retraction.

## Materials and Methods

### Bacterial Strains and Growth Conditions

Bacterial strains used in this study are listed in Supplementary Table 1. *M. xanthus* was grown in CTT medium (1% [w/v] casitone; 10 mM Tris–HCl, pH 7.6; 1 mM KH_2_PO_4_; 8 mM MgSO_4_) in the dark at 33°C with shaking. *E. coli* strains were cultured in LB media. As needed, 50 μg/ml of kanamycin (Km) was added to media. TPM buffer (CTT without casitone) was used to wash cells. For solid media, agar was added at 1.5 or 0.5%.

### Sequencing and Genetic Mapping

The genomes of DK1649 and revertants thereof were sequenced with the Illumina NextSeq 2000 platform (MiGS, Pittsburg, PA). To identify mutations the sequences were aligned against the WT DK1622 reference genome using BreSeq.

### Plasmid and Strain Construction

Plasmids and primers used in this study are listed in Supplementary Tables 2, 3. Gene disruptions were created by PCR amplification of 450–600 bp of internal gene amplicons and ligated into the pCR™-Blunt II-TOPO vector (Invitrogen), followed by electroporation into *E. coli* TOP10 cells and antibiotic selection. Constructs were verified by colony PCR, restriction analysis and sequencing, and subsequently used to transform *M. xanthus* by electroporation and selected for homologous recombination into the chromosome by Km resistance. To conduct rescue experiments, full-length WT genes were PCR amplified with high fidelity DNA polymerase Q5 (New England Biolabs) and ligated into pCR™-Blunt II-TOPO. After constructs were validated, they were electroporated into *M. xanthus* for homologous integration at the corresponding chromosomal locus. In-frame markerless deletions in *sglS* were made using a two-step homologous recombination method. Briefly, DNA fragments upstream and downstream of *sglS* were PCR amplified and cloned into the pBJ114 vector (Julien et al., 2000) using Gibson assembly (New England Biolabs). Verified constructs were electroporated into *M. xanthus*; transformants were selected for Km^r^ and subsequently counter-selected for plasmid excision on 2% galactose CTT agar plates (Ueki et al., 1996). Deletions were confirmed by PCR using primers flanking the deletion site and by phenotypic analysis. The GFP-SglS fusion was created by PCR amplification of msf*gfp* and *sglS* followed by Gibson assembly in-frame into pDP22 downstream of the *pilA* promoter. Verified plasmids were electroporated into a Δ*sglS* strain and selected for Km^r^. Transformants were confirmed by phenotypic analysis, western blotting and microscopy. The Δ*sglS-sglT*::Km double mutant was created by homologous recombination of a *sglT* insertion KO plasmid [pVT24, (Troselj et al., 2020)] into a Δ*sglS* strain by selecting Km^r^.

### Motility Assays

*M. xanthus* was grown in CTT overnight to mid-log growth phase, harvested, washed with TPM buffer, and adjusted to a cell density of 6 × 10^8^ cfu ml^–1^ and spotted on CTT with 1.5 or 0.5% agar to assess A- and S-motility, respectively. Micrographs were taken at various times following incubation at 24°C (room temperature) or 33°C (optimal growth temperature) using a Nikon E800 phase-contrast compound microscope or an Olympus SZX10 stereoscope coupled to imaging systems.

To determine the effect of temperature changes on S-motility, cells grown in CTT medium with 0.5 mM CaCl_2_ were spotted on CTT with 2 mM CaCl_2_ 0.5% agar-coated glass slides and incubated in a humid chamber at indicated temperatures and times. For time-lapse videos, culture inoculums were similarly dried on agar pads and viewed with an Olympus IX83 inverted microscope equipped with a 60 × oil immersion objective lens coupled to an Orca-Flash4.0 LT sCMOS camera.

### Congo Red Exopolysaccharide Binding Assays

To detect EPS, cells were grown overnight and resuspended in TPM to a density of 3 × 10^8^ cfu ml^–1^. Then 10 μl aliquots of cell suspension were spotted on CTT plates containing 30 μg ml^–1^ of Congo red dye and incubated for 5 days at 33°C, followed by imaging (Arnold and Shimkets, 1988).

### Trypan Blue Exopolysaccharide Binding Assay and Clumping Assay

Trypan blue binding assay was used to quantify EPS levels (Black and Yang, 2004). Briefly, strains grown in CTT to mid-log phase were harvested and resuspended in TPM. Aliquots of cell suspensions were mixed with 10 mg ml^–1^ trypan blue and TPM buffer was added to give a final cell density of 9 × 10^8^ cfu ml^–1^. For controls, cell free samples were used. After briefly vortexing, all samples were incubated for 30 min at 25°C in the dark. Cells were then sedimented, and the supernatants were transferred to cuvettes. The absorbance of each sample supernatant was measured at 585 nm. Absorbance values were compared to controls to quantify bound trypan blue.

A clumping assay was done to assess pili and EPS production (Arnold and Shimkets, 1988; Wu et al., 1997). Cells were grown in liquid CTT media overnight until they reached a mid-log phase. Cells were pelleted and resuspended in TPM buffer to a density of 9 × 10^8^ cfu ml^–1^ and transferred to glass tubes. Pictures were taken after a 1 h incubation at room temperature.

### Type IV Pili Shear Assay

To detect cell surface Tfp, a method adopted from Wall et al. (1998) was used. Briefly, sedimented cells from overnight cultures were washed, resuspended in TPM buffer to a density of 6 × 10^8^ cfu ml^–1^, and spotted on 1/2 CTT 2 mM CaCl_2_ 1.5% agar plates and incubated at 33°C for 3 h. Cells were then collected in 0.4 ml TPM buffer and pili were sheared off by vortexing at maximum speed for 2 min and separated from cells by centrifugation. Whole cell fractions were resuspended in 300 μl of SDS-PAGE sample buffer and heated at 95°C for 5 min. To precipitate pili fragments, 100 mM MgCl_2_ was added to supernatants, incubated in ice for 1 h, and then sedimented by centrifugation at 4°C at 20,000 × *g* for 20 min. The precipitated pili were resuspended in 30 μl of sample buffer and boiled for 5 min. All samples were stored at -20°C until used.

### Western Blot Analysis

SDS-PAGE was performed using the whole cell or sheared pili fractions. Primary rabbit antibodies used were anti-PilA (1:7,000; Wu and Kaiser, 1997) and anti-GFP (1:7,500; Invitrogen). Horseradish peroxidase conjugated goat anti-rabbit antibody was used for detection (1:15,000; Pierce). Blots were developed using SuperSignal West Pico Plus chemiluminescent substrate (Thermo Scientific) in a KwikQuant imager (Kindle Biosciences LLC).

### Transmission Electron Microscopy

For transmission electron microscopy, overnight mid-log phase cultures grown in CTT with 0.5 mM CaCl_2_ were centrifuged at 750 × *g* and washed gently with TPM buffer. Cells were carefully resuspended in TPM to the final density of 3 × 10^8^ cfu ml^–1^. A drop of cell suspension was pipetted onto a carbon-coated copper grid (FCF400-Cu, 400 mesh, Electron Microscopy Sciences). Cells settled for two min, and excess liquid blotted off. To visualize pili, a drop of 2% uranyl acetate (wt/vol) was pipetted onto the grid for 1 min and blotted dry. Transmission electron microscopy was done on a Hitachi 7000 instrument.

### Reversal Tracking

To determine the reversal frequencies of mutants grown at permissible temperature, cells were cultured overnight at 24°C as described above. After sedimentation and washing, cells were resuspended in TPM buffer to 2 × 10^8^ cfu ml^–1^ and spotted on CTT with 2 mM CaCl_2_ 0.5% agar-coated glass slides. As soon as the spots dried, 1 h time-lapse videos were made for single mutants and WT. However, the double mutant required an incubation for 5–6 h at 24°C in a humid chamber after agar pad spotting to restore some, albeit poor, motility. Movements of 20 isolated cells from each strain was manually tracked by time-lapse microscopy and reversal frequencies were plotted.

## Results

### Identification of the *sglS* Locus

**FIGURE 1:**
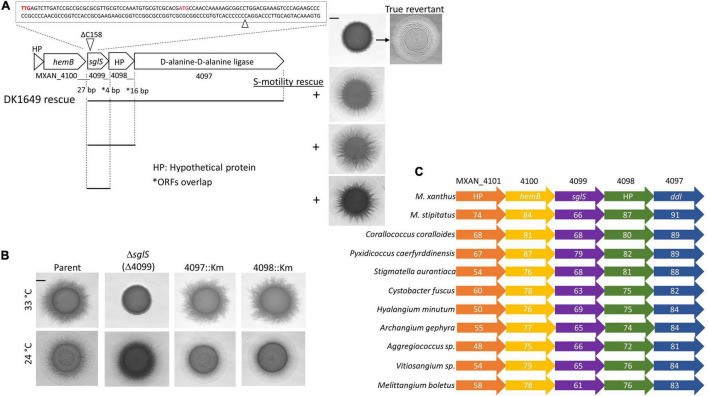
Genetic analysis of the *sglS* locus. **(A)** Sequence of *sglS*-1649 (MXAN_4099) region. The location of the 1 bp deletion is marked by triangles. Two alternative start codons highlighted in red. Cluster ORFs labeled with locus tags or gene names. Gaps or overlaps (*) between predicted reading frames indicated. Bottom, integrated DNA fragments (bars) used to rescue S-motility in DK1649 at 33°C (right). Top right, S-motility restored in a true revertant. **(B)** Isogenic strain set in an A^–^S^+^ parent strain (DK1217). Strains incubated at indicated temperatures for 72 h on soft CTT agar. **(C)** Conserved myxobacterial five-gene cluster encompassing *sglS*. The percent identity to DK1622 ORFs are listed inside each ORF. Scale bars, 2 mm. Strains described in Supplementary Table 1.

Prior large-scale screening campaigns done in the Kaiser laboratory isolated hundreds of S-motility mutants. These screens were done in strains that lacked A-motility and, importantly, chemical and UV mutagenesis was employed to ensure random, unbiased mutant isolation. Many mutations were mapped by classical methods to different loci, including a large *pil* gene cluster (Wu and Kaiser, 1997; Wu et al., 1997; Wall et al., 1999; Nudleman et al., 2006). However, a subset of mutations did not map to known loci suggesting they represented undiscovered S-motility genes (Wu, 1998). One S-motility mutant attracted our attention because it exhibited a *pilT*-like phenotype; cells made pili and clumped, thus implicating a role in Tfp function instead of assembly. This mutation was in strain DK1649, where it exhibited a temperature-sensitive (Ts^–^) S-motility phenotype (Wu et al., 1997). To identify the mutation, strain DK1649 was sequenced and compared to the wild-type (WT) DK1622 reference genome (Goldman et al., 2006). This analysis revealed many mutations that confounded gene identification. Fortuitously, however, we isolated three revertants of DK1649 that restored S-motility. These isolates were sequenced and were all found to contain a single base insertion in MXAN_4099, compared to the DK1649 genome, which restored the WT DK1622 sequence. These sequence changes resided in a stretch of seven cytosine (C) bases (Figure 1A). To validate these results we PCR amplified and re-sequenced this region and confirmed that DK1649 had a one base deletion and that our three isolates represented true revertants. Since DK1649 was originally isolated by ICR-191 mutagenesis, which causes insertion/deletion mutations, our findings were consistent with that mutagenic treatment, and the relatively frequent isolation of spontaneous revertants was also consistent with this polycytosine tract leading to slippage in DNA replication and base insertions (Farabaugh et al., 1978).

MXAN_4099 resides in an apparent operon with two downstream genes, MXAN_4098 and 4097, where these predicted ORFs overlaps. Therefore, to eliminate the possibility of polar effects on downstream genes, we inserted full-length copies of the genes at the native locus (rescue experiments in Figure 1A). The S-motility defect of DK1649 was successfully rescued with DNA fragments that covered MXAN_4097-4098-4099, MXAN_4098-4099 or only MXAN_4099. We conclude the S-motility defect in DK1649 was caused by a frameshift mutation in MXAN_4099, hereafter called *sglS* for *s*ocial *gl*iding (Hodgkin and Kaiser, 1979).

As mentioned, DK1649 was previously reported (Wu et al., 1997), and confirmed here, to contain a Ts^–^ S-motility defect. To clarify the nature of this phenotype and possible roles of the downstream ORFs in S-motility, we constructed mutations in each gene. To avoid complications associated with the other motility system, these mutations were made in strain DK1217 (*aglB1*, aka *aglQ1*), which lacks A-motility and is the parent of the reconstructed WT DK1622 strain (Dey et al., 2016). Insertion mutations in MXAN_4098 and MXAN_4097 did not affect S-motility and they exhibited no obvious morphological or growth defects, whereas the markerless in-frame Δ*sglS* mutation caused a conditional S-motility defect (Figure 1B). This result shows that the Ts^–^ phenotype was not result of a Ts^–^ protein; instead, SglS is conditionally required at high temperatures and that the *sglS*-1649 frameshift mutation also resulted in a null phenotype.

From bioinformatic analysis, *sglS* orthologs were exclusively found in myxobacteria. Strikingly, *sglS* and its four neighboring genes constitute a conserved gene cluster in myxobacteria and more specifically the Cystobacterineae suborder (Figure 1C; Cao et al., 2019). Sequence analysis revealed that SglS lacks a signal peptide and lacked a recognizable domain or protein fold. MXAN_4097 contains two-tandem D-alanine-D-alanine ligase (DDL) C-terminal domains (PF07478) implicated in peptidoglycan biosynthesis (Bruning et al., 2011). However, the primary *ddI* gene is likely MXAN_5601, which resides in a large gene cluster involved in cell wall biosynthetic and cell division. MXAN_4100 encodes a *hemB* ortholog involved in porphobilinogen synthase. MXAN_4097 and 4101 are hypothetical ORFs with undefined functions (Panek and O’Brian, 2002).

### SglS Null Mutant Exhibits a Reversible Ts^–^ Phenotype

**FIGURE 2:**
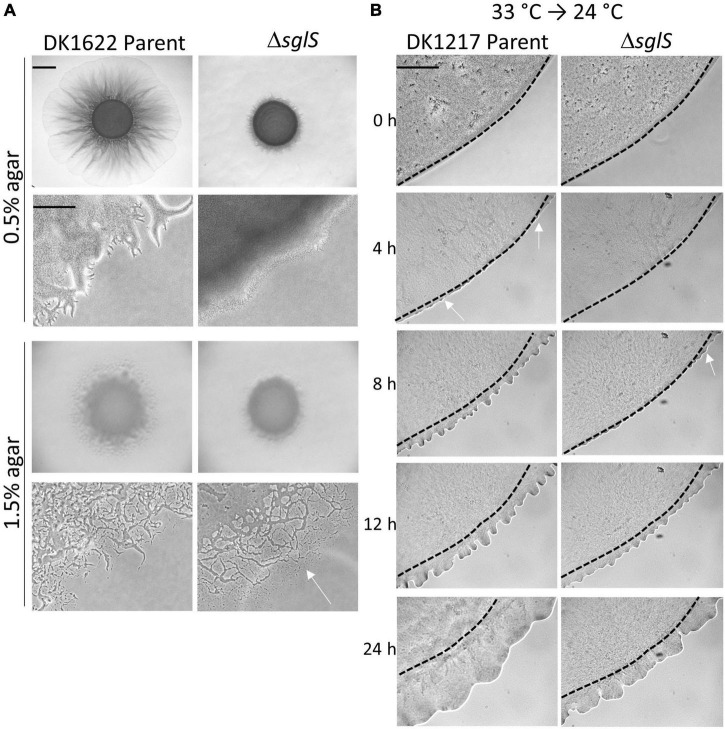
Phenotypic analysis of Δ*sglS* mutants. **(A)** Δ*sglS* mutation affects S-motility, but not A-motility in WT background (DK1622). Plates incubated at 33°C for 72 h. White arrow shows single cell migration indicative of A-motility. Scale bars, 2 mm and 200 μm. **(B)** ΔSglS mutant recovers S-motility following temperature downshift. Strains (*aglB1* background) grown in shaker flask at 33°C, spotted on CTT soft agar-coated slides and incubated at 24°C. Phase-contrast micrographs taken at indicated times. Representative micrographs shown from multiple replicates. Initial flare buds marked by white arrows, dashed-lines inoculum edge. Scale bar, 2 mm.

To investigate whether SglS plays a role in A-motility, we deleted *sglS* in the WT strain DK1622 that has A- and S-motility. The resulting mutant was incubated on hard agar and soft agar, which promote A- and S-motility, respectively (Shi and Zusman, 1993). In this WT background, only S-motility was found defective at 33°C on soft agar, whereas A-motility was unaffected (Figure 2A).

To investigate the time-course of temperature recovery, we grew liquid cultures of the Δ*sglS* mutant and its DK1217 parent strain at 33°C, and then transferred cells to soft agar-coated glass slides and further incubated at 24 or 33°C. Interestingly, initial signs of S-motility, as indicated by flare buds, appeared at around 8 h for the Δ*sglS* mutant and became prominent by 12 h (Figure 2B). This recovery period was more rapid than a SglT mutant (> 12 h; Troselj et al., 2020). In contrast, the parent strain showed emergent flares by 4 h. As expected, the Δ*sglS* mutant exhibited a more severe motility defect when agar pads were instead incubated at 33°C (Supplementary Figure 1). However, when cells were maintained at 24°C, the SglS mutant and parent strain showed emergent flares at the same time (Supplementary Figure 1). Additionally, to study individual cellular movements, we made high magnification time-lapse videos. Here, following the first hour of transfer from 33 to 24°C, ∼1% of the Δ*sglS* cells moved a cells length, whereas nearly all of the parent cells moved. In contrast, when transferred from 24 to 24°C, single cell movement was similar and robust for both strains (Supplementary Videos 1–4). Based on these results, we conclude that the SglS protein plays a critical role for Tfp-dependent motility at high temperatures.

### SglS Mutants Produce Exopolysaccharide and Pili

**FIGURE 3:**
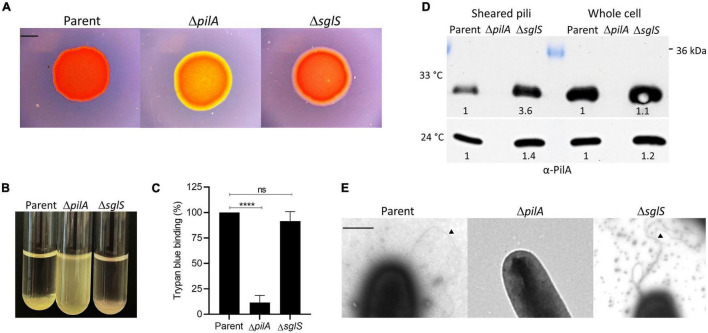
Exopolysaccharide and Tfp production by Δ*sglS* mutant compared to DK1217 parent. **(A)** Congo red binding where of PilA mutant serves as an EPS^–^ control (yellow-orange color). Scale bar, 2 mm. **(B)** Clumping assay where parent is a positive control and Δ*pilA* mutant is a negative control. **(C)** Quantitative assay of trypan blue binding to EPS. Percent dye bound calculated relative to the cell-free control and parent strain. **(D)** Pili shear assay where pilin (∼24 kDa) detected by immunoblot with α-PilA antibody at two temperatures. Pilin from whole cell fractions also shown. Band intensities measured by KwikQuant imager (Kindle Biosciences LLC) and relative intensities to parent shown. Representative blots shown where equal amounts of cell material was loaded. **(E)** TEM micrographs of cell poles and pili. Black triangles mark pili. Scale bar, 0.5 μm. Ns, not statistically significant; ****, *p* < 0.0001 as determined by unpaired *t*-test.

S-motility is powered by iterative cycles of Tfp extension and retraction. EPS also plays a crucial role serving as an extracellular matrix that holds cells together during group movement and as a trigger for pili retraction (Li et al., 2003). To qualitatively test for EPS production in a Δ*sglS* mutant, we conducted Congo red binding assays. After a 5-day incubation at 33°C, the mutant bound Congo red similar to the positive control, while a Δ*pilA* negative control did not (Figure 3A). We also conducted a cell-clumping assay, which indirectly measures EPS levels by its ability to aggregate cells from suspension (Wu et al., 1997). After 1 h incubation, the Δ*sglS* mutant clumped like WT whereas the Δ*pilA* mutant remained suspended in buffer (Figure 3B), again indicating the Δ*sglS* mutant produced EPS and pili. To measure EPS levels, a trypan blue dye-binding assay was employed. As shown in Figure 3C, the parent and the Δ*sglS* mutant produced equivalent levels of EPS in contrast to the Δ*pilA* negative control.

Next, since Tfp composed of PilA pilins drive S-motility, we investigated whether the Δ*sglS* mutant assembled extracellular pili. We used two approaches. First, we sheared and purified the long thin pili from whole cell surfaces by vortexing and fractionation followed by Western blot analysis. As shown in Figure 3D, when grown at 33°C, surface pili were present in the Δ*sglS* mutant at ∼3.6-fold higher levels than the parent strain, while the Δ*pilA* negative control strain produced no pili, indicating SglS mutants are hyperpiliated. In contrast, when grown at 24°C, the Δ*sglS* mutant produced shearable pili at near parent strain levels (∼1.4-fold higher). Secondly, we visualized pili by transmission electron microscopy and confirmed the presence of pili on cells in the Δ*sglS* mutant (Figure 3E). We therefore conclude, SglS mutants make EPS and are hyperpiliated at non-permissive temperatures.

### *SglS* Protein Produce Dynamic Cytoplasmic Puncta

**FIGURE 4:**
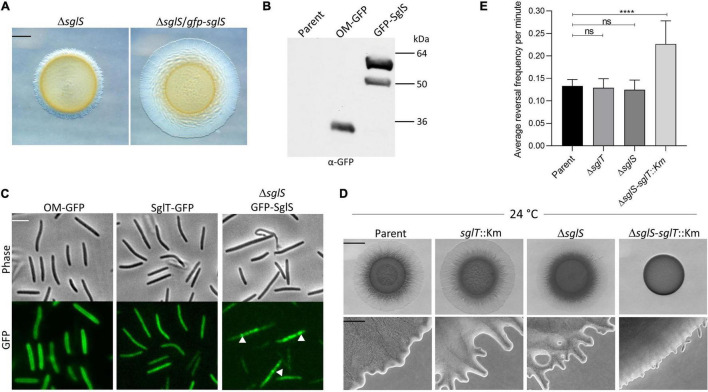
SglS and SglT localization and double mutant phenotype in DK1217 backgrounds. **(A)** The GFP-SglS fusion restores (complements) the S-motility defect caused by a Δ*sglS* mutation at 33°C on 0.5% agar. Scale bar, 2 mm. **(B)** Immunoblot shows the GFP-SglS fusion stably expressed at a predicted size of ∼50 kDa. Nature of higher mobility band unknown. OM-GFP is a lipoprotein fusion and serves as a control (Cao and Wall, 2017). Right, molecular weight markers. **(C)** Phase contrast and fluorescence microscopy images shows SglT-GFP (Troselj et al., 2020) uniformly localized in cytoplasm, while GFP-SglS forms puncta in the cytosol (white triangles), which are dynamic (see Supplementary Video 5). Scale bar, 5 μm. **(D)** Epistasis tests. Δ*sglS-sglT::*Km exhibits severe S-motility defect at 24°C, while single mutants exhibit WT motility. Plates incubated for 72 h. Scale bars, 2 mm and 200 μm. **(E)** Reversal frequencies averages for Δ*sglS-sglT*::Km double mutant (reversal frequency 0.2267; 1 reversal per 4.7 min) compared to single mutants *sglT* (0.1292; 7.9 min), *sglS* (0.1250; 8.2 min) and parent strain (0.1333 or 7.6 min). *Note, only ∼5% of the double mutant moved and were scored for reversals, while for the other three strains nearly all of the cells were motile. Each strain cultured at 24°C, 20 individual cell reversals were tracked based on corresponding Supplementary Videos. ns, not statistically significant (p > 0.05); ****, *p* < 0.0001 as determined by One-Way ANOVA test.

Tfp and many of its assembly proteins are located at the cell poles (Kaiser, 1979; Wall et al., 1999). Some of these proteins also undergo dynamic pole-to-pole oscillation when cells reverse their direction of movement (Bulyha et al., 2009). To investigate SglS cellular localization and possible dynamics, we constructed an N-terminal GFP fusion (monomeric superfolder variant). The fusion was functional because it complemented a Δ*sglS* mutation (Figure 4A). The fusion was also stably expressed at its predicted size (Figure 4B, ∼50 kDa; although a higher mobility band was also observed). Fluorescence microscopy revealed that GFP-SglS formed distinct puncta in the cell, which contrasted to the diffuse cytoplasmic localization of the SglT-GFP fusion (Figure 4C). High-speed time-lapse fluorescent microcopy also found that the GFP-SglS puncta were dynamic and moved back and forth in the cytoplasm (Supplementary Video 5). Upon visual inspection of time-lapse recordings, there was no apparent correlation with cellular motility or reversals and puncta movements. Therefore, the significances of the puncta and their dynamic movements remains unknown.

### Enhanced Synthetic Phenotype in a *sglT-sglS* Double Mutant

Since *sglS* and *sglT* mutants display similar Ts^–^ phenotypes, whereby they express WT levels of EPS and produce non-functional Tfp, indicated to us that these genes function in the same or related pathways. To address this question we conducted epistasis tests, where a double null mutant was made in the DK1217 background. Strikingly, the double mutant was severely defective in S-motility at low temperatures, which was in sharp contrast to either single mutant (Figure 4D). At high magnification, the double mutant only produced small colony edge flares after a three-day incubation. Time-lapse microscopy of the double mutant revealed that ∼95% of the cells did not move over the 30 min video and those that did reversed frequently and traveled short distances (Supplementary Video 6). From our videos, we measured the reversal frequencies of the double and single mutants alongside the parental control at 24°C (Supplementary Videos 3, 4, 6, and 7). Interestingly, for the minority of cells that sporadically moved in the Δ*sglS-sglT*::Km mutant, they reversed at twice the frequency as the parent strain and the two single mutants (Figure 4E). We conclude that since the single mutants each have similar phenotypes and the double mutant exhibits an enhanced synthetic phenotype, SglS and SglT function in parallel pathways, or perhaps the same pathway with overlapping functions, whereby at low temperatures only one protein is necessary for S-motility, while at high temperature both proteins or pathways are necessary.

## Discussion

We characterized a new S-motility gene, *sglS*, in *M. xanthus* and show it is conditionally required for Tfp-dependent motility and exhibits an enhanced synthetic phenotype with *sglT*. The original *sglS* mutation contains a frameshift mutation that results in a null Ts^–^ phenotype for S-motility, while at permissive temperatures motility is WT. SglS is not required for A-motility or growth, suggesting the mutation is not pleiotropic and that SglS plays a specific role in S-motility. At non-permissive temperatures, EPS production and pili assembly occur showing these processes are unaffected. Instead, SglS mutants actually assemble higher levels of shearable pili compared to parent cells, indicating SglS mutants are hyperpiliated. In contrast, at the permissive temperature, the level of shearable pili in the Δ*sglS* mutant are at WT levels. Based on these combined results we hypothesize SglS plays a specific role in Tfp function at elevated temperatures, likely involving the mechanical process of pili retraction. This is in contrast to EPS mutants, which are also hyperpiliated because they lack the signal for retraction, instead of a mechanical defect (Li et al., 2003; Hu et al., 2011; Saidi et al., 2021).

SglS is a hypothetical protein without a predicted function and its orthologs are exclusively present in the Cystobacterineae suborder of Myxococcales. Interestingly, *sglS* is found in a conserved gene cluster with four neighboring genes within Cystobacterineae. Among these genes are a predicted D-alanine-D-alanine-like ligase and a porphobilinogen synthesis, while the other two predicted ORFs are of unknown function. We further demonstrated that the two downstream genes are not required for S-motility, although these three ORFs do overlap and are in a conserved operon. Possible biological relationships between SglS and the other genes remain unknown.

We found that a GFP-SglS fusion protein formed dynamic cytoplasmic puncta, which unlike other Tfp proteins, are not localized at cell poles. The formation of puncta suggests SglS assembles into multimers, but the meaning on their dynamic oscillation is unclear.

Since SglS and SglT have similar phenotypes as PilT mutants, that is they all produce EPS, are hyperpiliated and non-motile (Wu et al., 1997; Black et al., 2006; Treuner-Lange et al., 2020; Troselj et al., 2020), suggests that SglS and SglT might facilitate PilT function in the retraction of pili at non-permissive temperatures. Alternatively, since a *pilT* null mutant in *M. xanthus* still exhibits residual retraction of Tfp (Clausen et al., 2009), other factors, including SglS-SglT, could be involved in a redundant or parallel pathway to PilT for pili retraction. Consistent with this, in other bacterial species PilU is also involved in pili retraction with PilT (Adams et al., 2019; Tala et al., 2019). For SglS and/or SglT, one possibility is that they act as a chaperone or assembly factor to form functional hexameric PilT/PilU retraction ATPases (Craig et al., 2019), particularly at elevated temperatures where protein aggregation is more problematic. In this regard, *sglS* and *sglT* could be heat shock genes, which warrants further investigation. Finally, we cannot exclude the possibility that these proteins function in another pathway. For example, O-antigen in lipopolysaccharide plays an undefined role in S-motility (Bowden and Kaplan, 1998; Perez-Burgos et al., 2019).

In contrast to either single mutant, the *sglS-sglT* double mutant exhibits an enhanced synthetic S-motility phenotype at low (permissive) temperatures, suggesting these proteins function in redundant pathways (Guarente, 1993; Forsburg, 2001). In this scheme, at low temperatures only one pathway is necessary for WT S-motility, whereas at high temperatures both the SglS and SglT are required. Finally, the high reversal frequency of the double mutant, albeit with greatly reduced motility where only a small fraction of cells move, indicates that their absence affects the coordination and/or signaling pathway that controls Tfp reversal frequencies (Spormann and Kaiser, 1999; Mauriello et al., 2010; Schumacher and Sogaard-Andersen, 2017). How this occurs is unknown, but a failure of pili to retract properly could interfere with reversal control.

## Data Availability Statement

The original contributions presented in the study are included in the article/Supplementary Material, further inquiries can be directed to the corresponding author.

## Author Contributions

KS performed experiments, data acquisition and analysis, and drafted the article. DW supervised experiments, assisted with data analysis and writing the manuscript. Both authors contributed to the article and approved the submitted version.

## Conflict of Interest

The authors declare that the research was conducted in the absence of any commercial or financial relationships that could be construed as a potential conflict of interest.

## Publisher’s Note

All claims expressed in this article are solely those of the authors and do not necessarily represent those of their affiliated organizations, or those of the publisher, the editors and the reviewers. Any product that may be evaluated in this article, or claim that may be made by its manufacturer, is not guaranteed or endorsed by the publisher.

## Supplementary Material

Supplementary Video 1 Time-lapse microscopy of S-motility in parent strain (DK1217, *aglB1*) that lacks A-motility at 33°C. Video made on 0.5% agar pad in CTT with 2 mM CaCl_2_ and spans 1 h with 20 sec frame intervals. Scale bar, 5 μm.

Supplementary Video 2 Time-lapse microscopy of Δ*sglS aglB1* mutant at 33°C as described for Supplementary Video 1.

Supplementary Video 3 Time-lapse microscopy of parent strain (DK1217, *aglB1*) at 24°C as described for Supplementary Video 1.

Supplementary Video 4 Time-lapse microscopy of Δ*sglS aglB1* mutant at 24°C as described for Supplementary Video 1.

Supplementary Video 5 Dynamic movements of GFP-SglS puncta in live cells. Time-lapse video for 2 min with 1 sec intervals between frames done on a glass slide. Scale bar, 5 μm.

Supplementary Video 6 Time-lapse microscopy of Δ*sglS-sglT*::Km *aglB1* mutant. Done as described for Supplementary Video 1 except soft agar pad incubated for 5 h at 24°C prior initiation of time-lapse for 30 min.

Supplementary Video 7 Time-lapse microscopy of *sglT*::Km *aglB1* mutant at 24°C as described for Supplementary Video 6.
