# Supplementary figures and images for: Conditional and Synthetic Type IV Pili-Dependent Motility Phenotypes in Myxococcus xanthus
Source: Front Microbiol. 2022 May 2;13:879090. doi: 10.3389/fmicb.2022.879090 (PMC9108774; doi:10.3389/fmicb.2022.879090)

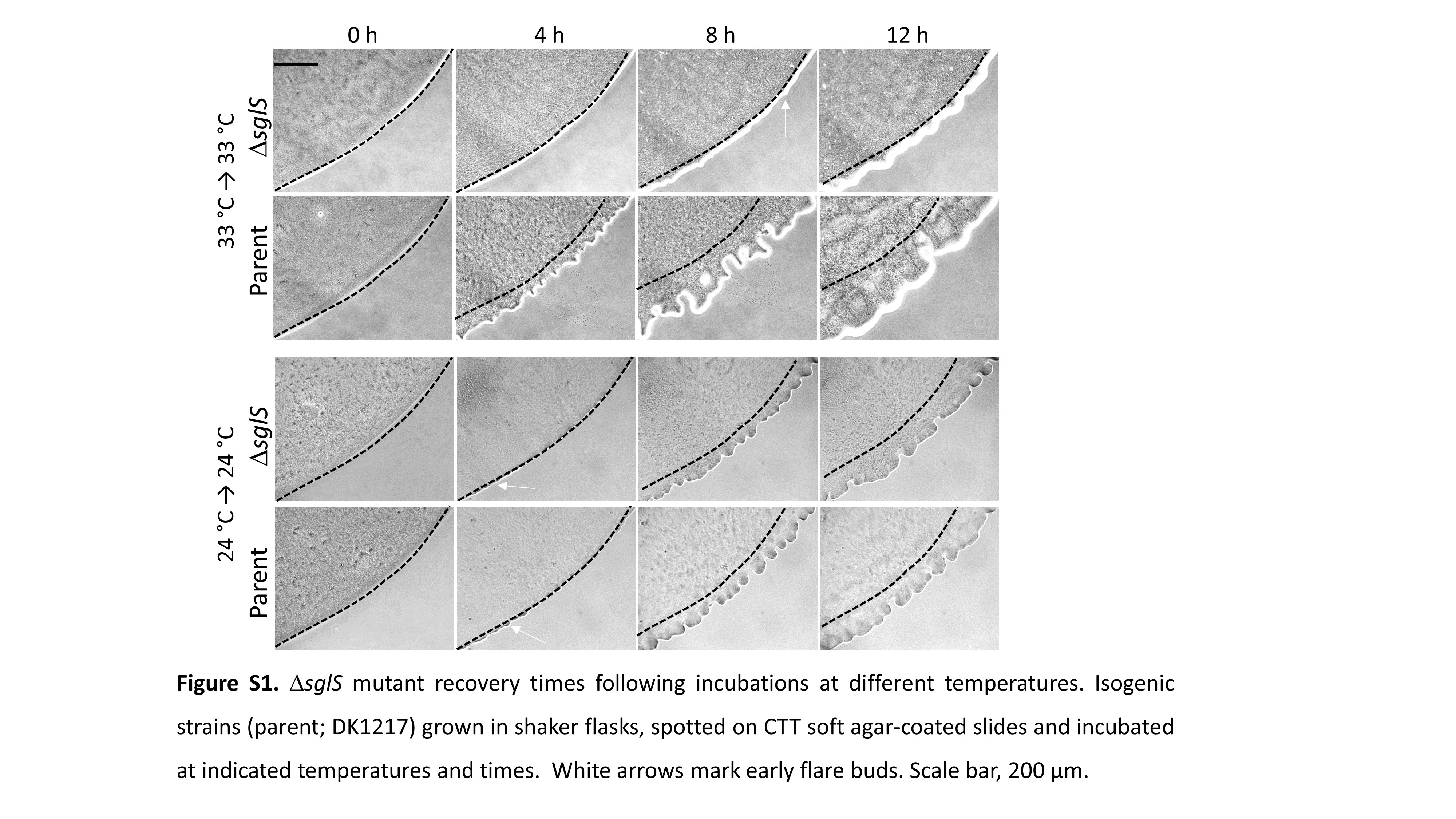

Supplement: Supplementary file 8 [file Image_1.JPEG]
