# Supplementary material for: Conditional and Synthetic Type IV Pili-Dependent Motility Phenotypes in Myxococcus xanthus
Source: Front Microbiol. 2022 May 2;13:879090. doi: 10.3389/fmicb.2022.879090 (PMC9108774; doi:10.3389/fmicb.2022.879090)
Supplement: Supplementary file 9 [file Table_1.docx]

**Table S1** Strains used in this study

| **Strain** | **Genotype** | **Source or reference** |
| --- | --- | --- |
| TOP10 | *E. coli* cloning strain | Lab collection |
| DK1622 | A^+^S^+^, WT *M. xanthus* | (Wall et al., 1999; Dey et al., 2016) |
| DK1217 | A^–^ S^+^, *aglB1* (aka *aglQ1,* F36→I) | (Wall et al., 1999; Dey et al., 2016) |
| DK8601 | A^–^ S^–^, Δ*pilA* *aglB1* | (Wall et al., 1998) |
| DK1649 | A^–^ S^−^, *cglC1* *sglS*-1649 | (Wu et al., 1997) |
| DW1800 | DK1649 4097-4098-4099-pCR-Blunt II-TOPO | This study |
| DW1801 | DK1649 4097-4098-pCR-Blunt II-TOPO | This study |
| DW1802 | DK1649 4097-pCR-Blunt II-TOPO | This study |
| DW1803 | DK1217 4097::pCR-Blunt II-TOPO | This study |
| DW1804 | DK1217 4098::pCR-Blunt II-TOPO | This study |
| DW1805 | DK1217 Δ*sglS* (markerless) | This study |
| DW1806 | DK1622 Δ*sglS* (markerless) | This study |
| DW1807 | DK1217 Δ*sglS sglT*::Km | This study |
| DW2704 | DK1217 *sglT*::pCR TOPO 2.1 | (Troselj, et al., 2020) |
| DW2275 | DK8601 SS^OM^-msfGFP | (Cao and Wall, 2017) |
| DW1808 | DW1805 P*_pilA_*-msf*gfp*-*sglS* | This study |
