# Supplementary material for: Conditional and Synthetic Type IV Pili-Dependent Motility Phenotypes in Myxococcus xanthus
Source: Front Microbiol. 2022 May 2;13:879090. doi: 10.3389/fmicb.2022.879090 (PMC9108774; doi:10.3389/fmicb.2022.879090)
Supplement: Supplementary file 10 [file Table_2.docx]

**Table S2** Plasmids used in this study

| **Plasmid** | **Relevant features** | **Source or reference** |
| --- | --- | --- |
| pCR-Blunt II-TOPO | Cloning vector, Km^r^, Zeo^r^ | Life Technologies |
| pDP22 | P*_pilA_* in pSWU19, Mx8 *attP*, Km^r^ | (Cao and Wall, 2017) |
| pBJ114 | Deletion cassette plasmid, Km^r^-Gal^s^ | (Julien et al., 2000) |
| pVT24 | *sglT* fragment in pCR TOPO 2.1, Km^r^ | (Troselj et al., 2020) |
| pKS1 | MXAN_4097-98-99 in pCR-Blunt II-TOPO, Km^r^, Zeo^r^ | This study |
| pKS2 | MXAN_4097-98 in pCR-Blunt II-TOPO, Km^r^, Zeo^r^ | This study |
| pKS3 | *sglS* in pCR-Blunt II-TOPO, Km^r^, Zeo^r^ | This study |
| pKS4 | MXAN_4097 KO fragment in pCR-Blunt II-TOPO, Km^r^, Zeo^r^ | This study |
| pKS5 | MXAN_4098 KO fragment in pCR-Blunt II-TOPO, Km^r^, Zeo^r^ | This study |
| pKS6 | *sglS* deletion cassette in pBJ114, Km^r^-Gal^s^ | This study |
| pKS7 | P*_pilA_-N-msfgfp-sglS* in pSWU19, Km^r^ | This study |
