# Supplementary material for: Conditional and Synthetic Type IV Pili-Dependent Motility Phenotypes in Myxococcus xanthus
Source: Front Microbiol. 2022 May 2;13:879090. doi: 10.3389/fmicb.2022.879090 (PMC9108774; doi:10.3389/fmicb.2022.879090)
Supplement: Supplementary file 11 [file Table_3.docx]

**Table S3** Primers used in this study

| Primer name | Sequence (5’ → 3’) |
| --- | --- |
| 4099_PCR_F | GCGGGTTGAGTCTTGATCCG |
| 4099_PCR_R | GCTTGCCGCCATGAACAC |
| 4098_PCR_F | GGAAGGACTCACGCTGGAG |
| 4098_PCR_R | CGAACTCCGCCTCTTCTTCC |
| 4097_PCR_F | CGCTCGTCGTTCAGGAAGC |
| 4097_PCR_R | GGAGGACACTAACCGACCG |
| 4099_SEQ_F | CCGGTCCACCGCGAAG |
| 4099_SEQ_R | GTTCCAGCCCTTGGCCG |
| 4097_TOPO_F | CATCGAGCGCATCGGC |
| 4097_TOPO_R | GCGACGTCTTCAGCGCA |
| 4098_TOPO_F | GCGCAACCCGATTCGC |
| 4098_TOPO_R | TGCAACGGTTACGCCCG |
| 4099_del_Frag1_F | CAATTTCACACAGGAAACAGCTATGACCATGATTACGCCAAGCTTCGCTCCGTCGGACATGATG |
| 4099_del_Frag1_R | CACTCCTCCGGCTCCAGCGTGTCCACCGTGGACAGCT |
| 4099_del_Frag2_F | TCGAGCTGTCCACGGTGGACACGCTGGAGCCGGAG |
| 4099_del_Frag2_R | GGGTTTTCCCAGTCACGACGTTGTAAAACGACGGCCAGTGAATTCTCTTCTTCCAAATCAGACAGCCTGAG |
| 4099_del_Fwd | CCCATCATGGCGTACTCGG |
| 4099_del_Rev | CCACGCTGCCGATGACA |
| 4099-N-msfGFP-F1 | CGTAGTGCTAATCCCATCCGCGGGTCTAGAAAGAAAGCGCGTAAGCGAATTAAAGGAGGTTTTTTATGAGTAAAGGTGAAGAACTGTTC |
| 4099-N-msfGFP-R1 | GACGCAACGCGCGCGGCGGATCAAGACTGCCGCCGCCGCCGCTTTTGTAGAGTTCATCCATGC |
| 4099-N-msfGFP-F2 | GCAGCAGGTATCACGCACGGCATGGATGAACTCTACAAAAGCGGCGGCGGCGGCAGTCTTGATCCGCCGCGC |
| 4099-N-msfGFP-R2 | GGGTTTTCCCAGTCACGACGTTGTAAAACGACGCCAAGCTTTCACTCCTCCGGCTCCAGCCTCCGGCTCCAG |

*F, Forward; R, Reverse. Restriction sites underlined.
